# A two-state comparative implementation of peer-support intervention to link veterans to health-related services after incarceration: a study protocol

**DOI:** 10.1186/s12913-017-2572-x

**Published:** 2017-09-12

**Authors:** Molly M. Simmons, Benjamin G. Fincke, Mari-Lynn Drainoni, Bo Kim, Tom Byrne, David Smelson, Kevin Casey, Marsha L. Ellison, Christy Visher, Jessica Blue-Howells, D. Keith McInnes

**Affiliations:** 1 VA Center for Health Organization and Implementation Research, Bedford, USA; 2 Boston University School of Public Health, Boston, USA; 3 Harvard Medical School, Boston, USA; 4 Boston University School of School of Social Work, Boston, USA; 5 VA National Center on Homelessness among Veterans, Bedford, USA; 6 University of Massachusetts Medical School, Worcester, USA; 7 VA New England Medical System, Bedford, USA; 8 University of Delaware, Newark, USA; 9 VA Healthcare for Re-Entry Veterans, Bedford, USA

**Keywords:** Facilitation, Vulnerable populations, Process mapping, Peer-support

## Abstract

**Background:**

Approximately 600,000 persons are released from prison annually in the United States. Relatively few receive sufficient re-entry services and are at risk for unemployment, homelessness, poverty, substance abuse relapse and recidivism. Persons leaving prison who have a mental illness and/or a substance use disorder are particularly challenged. This project aims to create a peer mentor program to extend the reach and effectiveness of reentry services provided by the Department of Veterans’ Affairs (VA). We will implement a peer support for reentry veterans sequentially in two states. Our outcome measures are 1) fidelity of the intervention, 2) linkage to VA health care and, 3) continued engagement in health care.

The aims for this project are as follows: (1) Conduct contextual analysis to identify VA and community reentry resources, and describe how reentry veterans use them. (2) Implement peer-support, in one state, to link reentry veterans to Veterans’ Health Administration (VHA) primary care, mental health, and SUD services. (3) Port the peer-support intervention to another, geographically, and contextually different state.

**Design:**

This intervention involves a 2-state sequential implementation study (Massachusetts, followed by Pennsylvania) using a Facilitation Implementation strategy. We will conduct formative and summative analyses, including assessment of fidelity, and a matched comparison group to evaluate the intervention’s outcomes of veteran linkage and engagement in VHA health care (using health care utilization measures). The study proceeds in 3 phases.

**Discussion:**

We anticipate that a peer support program will be effective at improving the reentry process for veterans, particularly in linking them to health, mental health, and SUD services and helping them to stay engaged in those services. It will fill a gap by providing veterans with access to a trusted individual, who understands their experience as a veteran and who has experienced justice involvement. The outputs from this project, including training materials, peer guidebooks, and implementation strategies can be adapted by other states and regions that wish to enhance services for veterans (or other populations) leaving incarceration. A larger cluster-randomized implementation-effectiveness study is planned.

**Trial registration:**

This protocol is registered with clinicaltrials.gov on November 4, 2016 and was assigned the number NCT02964897.

**Electronic supplementary material:**

The online version of this article (10.1186/s12913-017-2572-x) contains supplementary material, which is available to authorized users.

## Background

Justice reform, and subsequently, reentry services for individuals leaving incarceration have recently received increased attention from policymakers, media and the health services research community [1, 2]. Re-entry services are important because release from incarceration involves many risks for the former inmate. These include unemployment, homelessness, poverty, substance abuse (SUD) relapse and repeat offenses, particularly for persons with co-occurring mental illness and SUD [3–8]. In the first weeks post-release there may be gaps in mental health (MH) and SUD treatment while the individual tries to find a health care provider and renews medications [4, 6, 9–12]. Treatment engagement with primary care and mental health providers is essential to lowering the risk of mortality [13]. Without access to necessary outpatient health services, recently released individuals may rely on crisis-driven emergency room and hospital services. This may impede progress toward recovery and community integration, and may lead to re-offending and re-incarceration [3–7, 14].

The intersection of incarceration and mental illness particularly impacts veterans. Among veterans incarcerated in state prisons, 75% reported using drugs prior to incarceration, and roughly 25% of those reported injection drug use history [15]. About 50% of incarcerated veterans report having recently experienced symptoms of mental health disorders [15]. Veterans are more likely to report a recent history of mental health (MH) service use (30%) than non-veterans (24%) [15]. The Bureau of Justice Statistics reported in 2015 that 181,500 veterans were in jails and prisons in the United States during 2011–2012 [16]—about 8% of the incarcerated population. Between 12,000 and 56,000 veterans leave incarceration (hereafter “reentry veterans”) annually to transition back to the community [17].

Reentry services often begin prior to a person’s release and include making referrals to medical and MH services which individuals can access upon release. After release however, it can be challenging to facilitate or monitor individuals’ linkage to these services. In the Veterans Health Administration (VHA) reentry services are provided nationally by the Health Care for Reentry Veterans (HCRV) program [18]. HCRV outreach specialists work with incarcerated veterans to establish a post-release plan for linkage to VHA services [19]. The authorizing legislation that established HCRV emphasizes reentry planning in incarceration facilities rather than service delivery post-release [20]. The outreach specialists meet with veterans and make referrals for services such as housing, employment, legal assistance, primary care, MH, and SUD services. The authorizing legislation’s mandate, and the time and resource intensity of the in-facility reentry planning, means that the HCRV program has limited ability to follow reentry veterans to ensure that they attend the health care appointments to which they were referred. Internal VA data indicate 43% of reentry veterans do not have VA outpatient contact in the first 4 months post-incarceration, a time in which formerly incarcerated persons are particularly vulnerable to recidivism [20]. Linking to services that address some of the underlying risk factors, such as SUD and MH, may improve overall veteran health and contribute to economic well-being, and reduced recidivism rates (20).

Peer support represents a potential means of augmenting the HCRV program to cover a longer period of reentry, especially the first weeks after release. Peer support in this circumstance, sometimes called “forensic peers”, would likely be beneficial for a veteran population [21]. Peers with incarceration experience are likely to better understand and connect with veterans on a personal level than an outreach specialist who may not be a veteran or have incarceration experience [22]. Other peer programs have highlighted the benefits of using peers, and how they differ from the use of professionals [23]. First, peers tend to offer practical help; second, relationships between the peer and the recipient may involve self-disclosure and friendship; and third, peers can offer hope as a result of having experienced similar issues [24]. Studies of peer-support provided in prison environments, treatment courts, and to individuals under community corrections supervision (such as parole), have demonstrated effectiveness at reducing risk behaviors and improving health among justice-involved populations [25, 26]. Within the VA, peers could be trained to maintain contact with veterans, link them to VA health care services, and follow up with them to ensure they are going to follow-up visits [24, 25, 27]. The goal of our project is to enhance linkage to VA health and mental health services for veterans being released from incarceration through the use of a peer support system.

Our project will work with the national HCRV office to develop and implement an evidence-based peer support intervention. The intervention will extend the reach of the HCRV program through the peers who, for the first 6 months of the reentry period, will link veterans to VA health care services. We aim to design and implement the intervention in two states. Implementation science models and strategies are employed to increase the likelihood of success. The implementation science conceptual framework that guides this project is described below.

### Conceptual framework

The project is guided by the Consolidated Framework for Implementation Research (CFIR), a comprehensive and flexible framework that describes elements needed to achieve successful implementation. CFIR has five domains: Intervention Characteristics; Outer Setting; Inner Setting; Characteristics of Individuals; and Process of Implementation. Each is described below in relation to our study [28].

*Intervention characteristics* include constructs such as evidence strength and quality, adaptability, and complexity. There is growing evidence for the effectiveness of peer support in areas such as MH and SUDs in terms of improving linkage and engagement with services and enhancing outcomes [24–26]. This evidence is likely to assist in gaining support among stakeholders and providers for our peer support approach.

*Outer and Inner Settings* together comprise the context for an intervention. *Outer Setting* includes the economic, political and social context [28]. Importantly for our study of a highly vulnerable population, outer context includes individual needs and resources – i.e. the barriers and facilitators to health care access, utilization, and outcomes. For our population the barriers are likely to be considerable, given lack of economic resources, challenges to gaining employment, and the stigma of incarceration. Cosmopolitanism, a component of outer setting, refers to the extent to which an organization of interest is networked with external organizations. For this project these include state Departments of Corrections and Mental Health, and non-profit service providers. This deserves special attention from our project because the HCRV program straddles multiple delivery systems, including state prisons, VA services, and community organizations.

*Inner Setting* encompasses the structural, political, and cultural dimensions of organizations, including Networks and Communications which refers to the social, professional, formal and informal connections among providers within an organization [28]. For example, HCRV is under the purview of VA Homelessness Programs, but in any given VA medical center it also interfaces with other VA programs such as social work, primary care, and compensated work therapy. The level of communication and coordination among these programs will affect how well our target population’s needs are met.

*Characteristics of Individuals* refers to the targets of the intervention, i.e. clinicians, managers, and other health care personnel in the organizations providing services to reentry veterans. In our study, characteristics of individuals includes their knowledge and beliefs about the peer-support intervention, and their self-efficacy to adopt the intervention to achieve the implementation goals [28].

*Process of Implementation* includes Planning, Engaging, Executing, Reflecting, and Evaluating. Attention to this aspect will be essential for this project because it includes the intervention’s adaptation from one state to another. The Engaging component involves “attracting and involving appropriate individuals in the implementation and use of the intervention through a combined strategy of social marketing, education, role modeling, training, and other similar activities” [28]. We will use all these strategies, under an umbrella of Implementation Facilitation to implement this intervention (75). Description of our project’s use of Facilitation is in Methods, below.

### Project aims

The goal of this project is to implement and evaluate a peer-support program for reentry veterans. The specific aims are:

1. Conduct contextual analysis to identify VHA and community reentry resources, and to describe how reentry veterans use them.
2. Implement peer-support intervention, in one state, to link reentry veterans to Veterans Health Administration (VHA) primary, MH, and SUD services. We will use external and internal facilitation as the implementation strategy. Evaluation will include intervention fidelity, linkage to VA health care and utilization of health care services.
3. Adapt and implement the peer-support intervention in another, geographically, and contextually different state. At the end of this study, we will develop a multi-region study, using a hybrid type III, stepped-wedge design, to evaluate implementation across multiple geographic and contextual settings.

## Methods

This peer support intervention involves a 2-state sequential implementation study (Massachusetts, followed by Pennsylvania) using a facilitation implementation strategy. Below we describe the use of Facilitation as our implementation strategy and then we describe the peer supported intervention. This is followed by the description of the three phases of our project (each lasting roughly 12 months). Table 1 summarizes our project methods and timeline. The World Health Organization (WHO) Trial Registration Dataset, included as an Additional file 1, contains information about the project organization and study design. A populated SPIRIT checklist is also included in Additional file 2.

**Table 1:** Overview of study design, data collection, and analysis

	Phase 1: contextual analysis of reentry environment and resources in Massachusetts	Phase 2: Massachusetts implementation of peer support	Phase 3: Pennsylvania implementation of peer support
Study population	• 10 veterans released from incarceration • 20 stakeholders who currently assist with reentry services	For intervention with veterans released from incarceration: • 30 veterans for intervention group • 60 veterans for comparison group	• For formative work: 10 veterans released from incarceration and 15 stakeholders • For intervention with veterans released from incarceration: 30 intervention veterans and 60 comparison group veterans
Recruitment of veterans	• Reentry outreach specialists approaching veterans they have recently served, and approaching veterans in prisons prior to their release	• Reentry outreach specialists approaching veterans prior to their release	• Reentry outreach specialists approaching veterans prior to their release
Data Sources, and Collection	• Veterans will each be interviewed 3 times (at 1-week, 1-month, and 6-months post-release) • Stakeholders will be interviewed once.	• Health care utilization questionnaire with intervention veterans at baseline (week 1) and 6 months to capture information about VA and non-VA health service use. • Interviews with stakeholders and veterans. • Internal VA clinical and administrative data to compare intervention and comparison veterans	• For contextual analysis an abbreviated version of Phase 1 in Massachusetts: veterans interviewed 2 times over a six-month period and stakeholders will be interviewed once. • For implementation: same as Phase 2 in Massachusetts (health care utilization questionnaire, interviews, etc.)
Analysis	• Contextual network mapping and thematic analysis using grounded codes and a priori codes.	• Compare rates of visits for primary care, MH, SUD, no-shows between intervention and comparison groups; t-tests and chi-square tests. • For secondary measures: compare ER and hospital use (episodes and number of days)	• Same as for Massachusetts Phase 2 implementation of peer support
Outputs	• Network map to show reentry services, processes for linkage and delivery of services, and gaps in services	• Peer support guidebook • Peer support training curriculum	• Network map similar to approach used in Massachusetts • Peer support guidebook and training curriculum adapted for Pennsylvania
Timing	• Months 1–12 of trial	• Months 12–24 of trial	• Months 20–36 of trial

### Implementation strategy: Facilitation

In keeping with the “engaging” component of the CFIR framework we selected Facilitation as our strategy to promote adoption and use of the intervention. Facilitation is a comprehensive implementation approach in which implementation researchers partner with local staff to support implementation planning and to tailor adoption strategies to the local context [29, 30]. Facilitation has been used extensively as an implementation strategy in VHA [31]. External facilitators (EFs) bring external expertise in implementation processes and have transferable knowledge in relevant clinical and behavioral models that inform intervention; an internal facilitator (IF) is a member of the organization where the intervention is being implemented and is familiar with facility-level organizational structures, procedures, and culture. Facilitation will include training, ongoing problem solving and technical assistance provided to HCRV managers, outreach specialists, and peers over the course of the project.

It is not known what organizational position is best suited to facilitation of this intervention, thus we will examine two different levels. In Massachusetts the internal facilitator will be one of the HCRV outreach specialists; that is at the lowest organizational level. In Pennsylvania the internal facilitator will be one of the two Veterans’ Integrated Service Network (VISN)-level homelessness coordinators, a position at a higher organizational level that supervises multiple outreach specialists. A VISN is a regionally defined service area within the VHA which typically covers multiple states. Massachusetts is in VISN-1 while Pennsylvania is in VISN-4. The placement of the IF at a higher organizational level in Pennsylvania will help determine which level of internal facilitation is best for this kind of state implementation.

### Peer-support intervention

The peer support intervention will include a set of core elements for replicability to ensure fidelity of implementation, while also being flexible for adaptation to the specific context. Existing peer support models will be examined to identify evidence-based elements for inclusion, as well as to identify training and support components to incorporate; other aspects of our intervention will be newly developed based on lessons from the contextual analysis conducted as part of phase 1 of the study (details on phases to follow below).

Recent interventions using peers with vulnerable veterans provide some guidance to the current intervention. These include the Maintaining Independence and Sobriety through Systems Integration, Outreach and Networking (MISSION) intervention used with veterans with co-occurring mental health and substance use disorder [22], VetSEd, a peer-based supported education initiative for veterans [32–35], and a low-intensity peer and navigator approach to linking and engaging homeless veterans to health care [36].

Intervention design will also draw from a range of re-entry programs developed for non-veteran populations. While many programs focus on working with offenders during incarceration, we will focus on interventions that engage with the offender at the time of discharge and beyond. These include the Critical Time Intervention (CTI) [37], Support Matters [38] and Project Bridge [39].

### Key outcomes

Our primary measures to asses fidelity to the intervention are number of peer contacts attempted and made with each veteran, number of months in the 6-month intervention period in which a veteran had at least 1 peer contact, and proportion of guidebook material covered by the peers with each veteran. These measures will be assessed through a Peer Encounter Workload Form adapted from Ellison, which is completed by the peer and includes his/her weekly delivery of service, the types of contacts (in person, phone) and the content discussed (navigating VHA, logistics of appointments, etc.) [35].

The veteran level outcomes to measure linkage to VA services and utilization of health care services include: number of primary care visits, MH visits, and SUD visits; and missed opportunity rates (no-shows and cancellations) for primary care, MH, and SUD visits. Secondary outcomes include number of emergency room (ER) visits, hospitalizations, and hospital days.

### Procedures

Below we describe procedures and specific details about each of the study’s 3 phases.

#### Phase 1: Contextual analysis of Reentry environment and resources in Massachusetts

Phase 1 starts with contextual analysis [40–42] through interviews and network mapping of organizations and reentry services in Massachusetts. At the end of this phase the findings will be incorporated into the peer-support guidebook. This phase will involve interviewing Veterans and stakeholders. Network mapping is a technique used in business and engineering, and more recently in health care. It involves documenting potential paths that individuals and groups follow to accomplish a task (here, linkage to primary care and other health services) [43–45]. The timing, sequence, and duration of veterans’ contacts with individuals and organizations will be captured in activity diagrams [44], which can be combined to create a comprehensive map of the network of services and the reentry-related contacts veterans make. Such maps may reveal barriers to successful reintegration including unhelpful contacts, long waiting times between contacts, and useful but infrequently tapped resources. We will not incorporate Pennsylvania into the study until the end of phase 2.

### Study population

The study population includes the 100–150 veterans released from Massachusetts prisons each year. A sample will be drawn from this population. We will also sample from stakeholders who are leaders, managers, and service providers in organizations serving reentry populations.

### Recruitment of veterans

During prison visits, the outreach specialists will collect contact information from incarcerated veterans who would like to participate in contextual analysis phase of the project after their release. The outreach specialist will provide our team with the contact sheets of those veterans and their release dates. We will contact Veterans after their release, with the goal of conducting qualitative interviews with 10 participants, a number sufficient for thematic saturation [46, 47].

### Inclusion and exclusion criteria

Veterans released from a Massachusetts state prison, eligible for VHA services, and with no history of dementia or other serious cognitive impairment that would interfere with their being interviewed.

### Data sources, collection, and analysis

Veterans will each be interviewed 3 times (at 1-week, 1-month, and 6-months post-release), and stakeholders will be interviewed once. Interview questions will be guided by the CFIR framework and previous literature on linking and engaging vulnerable populations in health and social services, such as the Behavioral Model for Vulnerable Populations [48]. We will place a special emphasis on inquiry about patient needs and resources and will also ask questions about enabling characteristics and health-related need based factors. As indicated above, the interviews will also involve questions to create activity diagrams and network maps. For further information, see the draft interview guides in Additional file 3 in the Additional Materials section. Veterans will receive a $25 store gift card for completion of each interview.

### Stakeholder interviews

Participants will include leaders, managers, and providers, in VA (e.g. primary care, MH, SUD, homeless programs) and in non-VA organizations such as programs for persons with justice involvement, health care for homeless programs, hospital ERs, and state departments of corrections.

### Interview analysis

Interviews will be transcribed verbatim from the audio recordings, and analyzed using NVivo, a qualitative data analysis software [49]. Three qualified coders (JH, RB, MS) from our team will separately code transcripts using a-priori coding based on CFIR and literature relating to vulnerable populations needs, services, linkage, and engagement [50, 51]. Following grounded theory methods we will also identify themes that emerge directly from the data that help us understand issues related to linkage and engagement in health care. A VA systems engineer (BK) will facilitate the project team’s generation of network maps and then the research team will combine interview and network map findings to create two descriptions of reentry resources: from the perspectives of veterans and reentry stakeholders, respectively. We will use member checking [52] to check the accuracy of the network map with interview participants. This information will guide the content of the peer-support intervention, and will be incorporated into the peer-support guidebook.

#### Phase 2: Massachusetts implementation of peer support & preparation for Pennsylvania implementation

This phase involves implementation and evaluation of the peer-support intervention in Massachusetts, and preparation for initiating the intervention in Pennsylvania. Study population, recruitment of veterans, and inclusion/exclusion criteria will be the same as for Phase 1 except that in this phase we will recruit veterans while they are still incarcerated instead of after release. This is to create rapport between the veteran and the peer before the veteran is back in the community. The veterans will be identified by the outreach specialist and will have expressed interest in taking part in this study. Our target is 30 veterans receiving peer support. We will use the VA’s Homeless Operations Management and Evaluation System (HOMES) database, which includes incarceration-related data on veterans, to create a 2:1 matched comparison group of 60 veterans released in the same time period in Massachusetts. Matching will be conducted via propensity scores, and will be based on demographics (i.e. age, race/ethnicity, gender), SUD/MH diagnoses, criminal offense, length of incarceration, and number of arrests. The intervention will last 6 months for any single veteran, with peers having caseloads of 15 veterans [53]. A VISN 1 outreach specialist will serve as internal facilitator, while two members of our study team (KM, MM) will be external facilitators.

### Data sources, collection and analysis

Peers will administer a health care utilization questionnaire with intervention veterans at baseline (week 1) and at 6 months to capture information about VA and non-VA health services use.

During the Massachusetts implementation we will collect formative evaluation data about the intervention through interviews (5 stakeholders and 5 veterans). We will assess elements of the implementation by learning what meanings the participants (stakeholder, peers, veterans) assign to the intervention and the processes that the intervention is designed to affect [54]. We will also conduct summative evaluation, including qualitative interviews (5 veterans and 5 stakeholders) to evaluate, for example, if peers were enabling linkage to and engagement in health care, and whether this helped address veteran’s health care needs. With stakeholders we will explore the effectiveness of external and internal facilitation, and identify facilitators and barriers to implementation.

Our quantitative analysis will be based on data obtained from VA electronic medical records, available in the VA’s Corporate Data Warehouse [55]. We will use these data to compare Veterans in the intervention and matched comparison group with respect to our primary outcomes measures of the rate and volume of visits for primary, MH, and SUD outpatient care, as well as missed opportunities for outpatient care (i.e. appointment no shows) in the 6-month reentry period. We will also use VA electronic medical record data to construct our secondary outcome measures: the number of emergency department visits and inpatient hospitalization days during the 6-month reentry period. Analysis of these outcome measures will be conducted in two phases. First, we conduct bivariate comparisons of Veterans in the intervention and matched comparison group on the primary and secondary outcome measures using either t-tests or chi-square tests, depending on the distribution of the outcome measure. Second, we will use regression models to estimate the relationship between membership in the intervention group and each of the outcome measures, after adjusting for relevant covariates including age, race/ethnicity, gender, SUD/MH diagnoses, criminal offense, length of incarceration, number of arrests, and any prior history of VA health service use, as identified in VA electronic medical records. The exact functional form for each of the regression models will depend on the distribution of the outcome variable in question (e.g. logistic regression for binary outcomes, ordinary least squares for continuous outcomes, Poisson/negative binomial regression for count outcomes).

We will conduct additional exploratory analysis of outcomes specifically among Veterans in the intervention group. We will use paired t-tests and data obtained from the peer administered health care questionnaire to conduct a pre-post comparison of use of VA and non-VA health services use at baseline (week 1) and 6-months. We will also assess the bivariate relationship between the fidelity measures (i.e. number of peer contacts with veterans, number of months in which veteran had at least 1 peer contact, and proportion of guidebook material covered) and the 6-month measures of use of VA and non-VA health services using parametric and non-parametric tests for correlation (i.e. Pearson’s R^2^, Spearman’s ρ) as appropriate. We have not yet started to collect data for this phase.

#### Phase 3: Pennsylvania contextual analysis and implementation of peer support

Concurrent with the end of Phase 2 in Massachusetts, we will conduct contextual analysis of Pennsylvania’s reentry environment using methodologies similar to those used in Massachusetts. We will do this in preparation for implementation of the intervention in Pennsylvania. The process in Pennsylvania, however, will be briefer because we will have learned lessons from Massachusetts that will allow us to adapt to individuals and issues that are specific to Pennsylvania, without needing to cover as many of the general issues that confront all veterans in reentry generally. We anticipate in Pennsylvania conducting only 2 interviews for each of the 10 veterans, and single interviews with up to 15 stakeholders. Network mapping will be used as part of contextual analysis to describe common pathways that veterans take during the reentry process.

The peer support guidebook developed for Massachusetts will be adapted to Pennsylvania based on contextual differences between Massachusetts and Pennsylvania. The recruitment of veterans will be very similar to what was described in Phase 2 for Massachusetts. Peer training will be conducted in person in Pennsylvania by the same training team as for Massachusetts (the location will be determined during contextual analysis based on input from Pennsylvania stakeholders).

### Study population

Pennsylvania has paroled and released 130 Veterans from their Veterans Service Units in the last 3 year. The sample will be drawn from this population. As in Massachusetts, our goal is to recruit 30 veterans for the intervention group and a matched group of 60 for our comparison group. Veterans and stakeholders, in similar numbers to Massachusetts, will also be interviewed for formative evaluation and summative evaluation.

### Data sources, collection, and analyses

The data sources, collection techniques and analysis will be largely the same as for Massachusetts. One difference for Pennsylvania is that our interviews will assess how well the intervention was adapted to Pennsylvania. We will use the same internal VA data sources used in phase one to assess whether there are differences between the intervention and comparison veterans with respect to the primary and secondary outcome measures. In addition, we will pool data from both the Massachusetts and Pennsylvania sites to compare veterans in the intervention and matched comparison groups with respect to the primary and secondary outcomes described above. We will also use pooled data to conduct analysis of veterans in the intervention group assessing the relationship between the intervention fidelity measures (i.e. number of peer contacts with veterans, number of months in which veteran had at least 1 peer contact, and proportion of guidebook material covered) and the 6-month measures of use of VA and non-VA health services.

### Sample size and power

This project is intended to evaluate the feasibility of implementing peer-support for reentry, with the aim of informing an eventual larger scale cluster-randomized hybrid implementation-effectiveness study that will allow for a more rigorous assessment of the impact of the intervention. Thus, our sample size is driven primarily by practical issues related to the number of peers that it will feasible to train at each site and the caseload size it was deemed feasible for each peer to manage rather. Consequently, anticipated sample will have adequate power only to detect medium to large effect sizes in all of our site-stratified quantitative analysis. Meaning, with anticipated sample size of 30 Veterans in the peer-support intervention group and 60 veterans in the matched comparison group at each site, a comparison of the proportion of Veterans in the intervention and comparison group at each site accessing primary care would have a minimum detectable effect size (Cohen’s *h*) of .63, setting statistical significance at the 0.05 level and assuming 80% power. This effect size is considered to be between medium (*h =* 0.5) and large (*h =* 0.8) by conventional standards and would be equivalent to increasing the linkage rate, now at 57%, to about 85%. Analysis across including veterans from both sites will have greater power; a comparison of the proportion of veterans in the intervention and matched comparison group accessing primary care would be able to detect an effect size (Cohen’s *h*) of .44, which is considered to be between small (*h* = 0.2) and medium (*h =* 0.5). To address limitations related to sample size and power, we will report 95% confidence intervals around all effect sizes.

## Discussion

Reentry veterans are a particularly vulnerable population—at high risk for homelessness, morbidity, mortality and recidivism. Engagement with needed services can help combat serious health issues. This intervention provides a path for that engagement through the use of peers who more fully understand the experience and difficulties facing reentry veterans. This study will contribute valuable information in a number of different domains: 1) it will provide the first data on feasibility of reentry peer supported interventions in VA, helping to broaden the evidence base for this approach beyond its current use with patients with mental health, substance use, and employment who are not re-entry veterans [37, 38]. 2) It will also contribute to implementation science in demonstrating the value of network mapping as a tool for formative work when implementation depends on multiple organizations and systems providing services to the same population [56]. 3) It will test two different modes of internal facilitation – in one setting it is an outreach worker who is a service provider in the HCRV program, while in the other setting it is a higher level (organizationally) that is the VISN homelessness program coordinator. 4) The network mapping process and the final understanding of the flow of reentry veterans through contacts with systems, organizations, and individuals will make explicit what may be poorly understood processes, even for long-time providers and stakeholders in the realm of reentry services. 5) It will also help identify for peers and other providers the most important leverage points, and also the weakest transitions e.g. from one provider or organization to another. Last, the intervention will integrate findings from the network mapping to design a robust peer-support training program. This approach will allow us to more effectively target our resources and refine implementation approaches to help with sustainability in the 2 study sites. It will also lead to a process for adaptation and spread to other states.

This project comes at a critical time for policy regarding reentry programs and services. Not only is the need great but there is increased attention to this issue. President Obama focused the media and policymakers on this issue when he made criminal justice reform a key issue in his final year in office [1]. The Senate Judiciary Committee also recently passed legislation out of committee that, should it become law, would address some of the difficulties experienced by individuals reintegrating into society following imprisonment [57]. These reforms could potentially increase the flow of persons leaving incarceration, thus creating some urgency to improve the reentry services for this expanding population. The findings from this project can therefore help to improve services at this critical time.

This project is not without its limitations. With a small sample size and a non-randomized design there is limited ability to detect the effect of the intervention on linkage and engagement rates. However, the small sample size also makes it possible to conduct detailed network mapping as part of our contextual analysis. The non-randomized design also means that if associations between peer support and the processes and outcomes of interest are found, they may be biased due to unmeasured confounding. The intervention takes place in only two states, in the eastern United States, and thus the findings may not be generalizable to other states. But, the two state design does provide a clear case study of applying experience from one state to implementation in another. This can help eliminate redundancies in designing the eventual larger multi-site implementation trial. Finally the sample will include veterans only, which will be an overwhelmingly male population. Thus findings may not be applicable to non-veterans or to women. The next phase of our research, however, will address many of these limitations. We plan to conduct a large cluster-randomized hybrid implementation-effectiveness study spanning several states representing diverse geography and socio-economic status.

## Conclusions

Reentry veterans may experience a plethora of pitfalls as they navigate reentry and reintegration into their communities. Based on evidence from other health and social service programs where peers are used, we anticipate that the proposed peer support intervention will be effective in improving the reentry process for veterans. Peers may help reentry veterans overcome mistrust of VA and other organizations, mistrust that may have been bred from past negative experiences with large organizations and systems. Our project will help demonstrate whether reentry peer support is feasible, and contributes to veterans’ linkage to health care, MH care, and SUD care after incarceration. It will provide a roadmap – in the form of guidebook, training materials, implementation strategies, and evaluation results – so that other states can efficiently adapt and implement this approach in their HCRV programs. The anticipated linkage and engagement will contribute to improved health, MH, and reduced addictive behaviors of the thousands of veterans released from incarceration annually, helping to prevent the cascade of events that lead to negative behaviors, re-offending, and re-incarceration.

## Additional files

Additional file 1: WHO Trial Registration Dataset. (DOCX 20 kb)

Additional file 2: SPIRIT Checklist. (DOC 100 kb)

Additional file 3: Interview Guides. (DOCX 25 kb)

## Abbreviations

CIFR: Consolidated framework for implementation research
HCRV: Healthcare for re-entry veterans
MH: Mental health
SUD: Substance use disorder
VA: Department of Veterans Affairs
VHA: Veterans Health Administration
