# Supplementary material for: A two-state comparative implementation of peer-support intervention to link veterans to health-related services after incarceration: a study protocol
Source: BMC Health Serv Res. 2017 Sep 12;17:647. doi: 10.1186/s12913-017-2572-x (PMC5596492; doi:10.1186/s12913-017-2572-x)
Supplement: Supplementary file 1 — WHO Trial Registration Dataset. (DOCX 20 kb) [file 12913_2017_2572_MOESM1_ESM.docx]

**Additional file 1: World Health Organization Trial Registration Dataset**

| **Data Category** | **Information** |
| --- | --- |
| Primary registry and trial identifying number | Clinicaltrials.gov  NCT02964897 |
| Date of registry in primary registry | November 4, 2016 |
| Secondary identifying number | Not Applicable (NA) |
| Sources of monetary or material support | Grant funding: “Bridging the Care Continuum” (QUE 15-284); and  VA Center for Innovation post-doctoral fellowship |
| Primary sponsor | Department of Veterans Affairs, QUERI Program  Contact information: amy.kilbourne@va.gov |
| Secondary sponsor | NA |
| Contact for public queries | Keith McInnes, ScD  Edith Nourse Rodgers Memorial VA Hospital  200 Springs Rd.  Bedford, MA 01730  (718) 687-3507  Keith.mcinnes@va.gov |
| Contact for scientific queries | Keith McInnes, ScD  Edith Nourse Rodgers Memorial VA Hospital  200 Springs Rd.  Bedford, MA 01730  (718) 687-3507  Keith.mcinnes@va.gov |
| Public title | A Two-State Comparative Implementation of Peer-Support Intervention to Link Veterans to Health-Related Services after Incarceration: a Study Protocol |
| Scientific title | Improving Linkage to Health and Other Services for Veterans Leaving Incarceration (PIE) |
| Health condition(s) or problem(s) studied | Linkage to Care Homelessness Substance Use Disorder Mental Health |
| Interventions | Intervention: Peer-support plus usual care from the Health Care for Reentry Veterans program Control: Usual care from the Health Care for Reentry Veterans program |
| Key inclusion and exclusion criteria | Inclusion:   - Veteran released from a Massachusetts state prison - Veteran released from a Pennsylvania state prison - Exclusion: - A history of dementia or other serious cognitive condition that would prevent them from being interviewed or completing a survey questionnaire. |
| Study type | Interventional pilot implementation study  Allocation: Non-Randomized Intervention Model: Parallel Assignment Masking: No masking Primary Purpose: Supportive Care |
| Date of first enrollment | September 2017 |
| Target sample size | 120 |
| Recruitment status | Haven’t started recruiting, conducting formative evaluation interviews |
| Primary outcome(s) | - Linkage to healthcare services [ Time Frame: 6 months after release from incarceration ]   Participant will have made and kept one or more primary or specialty health care appointments. |
| Secondary outcome(s) | - Living in transitional or permanent housing [ Time Frame: 6 months after release from incarceration ]   Subject is residing in transitional or permanent housing. Transitional housing is housing that provides appropriate supportive services (e.g. MH or SUD group therapy) to recently homeless persons to help them achieve independent living. Transitional and permanent housing do not include emergency shelters, living on the streets, or living in a structure unfit for human habitation. |
