# Supplementary material for: A two-state comparative implementation of peer-support intervention to link veterans to health-related services after incarceration: a study protocol
Source: BMC Health Serv Res. 2017 Sep 12;17:647. doi: 10.1186/s12913-017-2572-x (PMC5596492; doi:10.1186/s12913-017-2572-x)
Supplement: Supplementary file 3 — Interview Guides. (DOCX 25 kb) [file 12913_2017_2572_MOESM3_ESM.docx]

**Additional file 3: Interview Guide**

Veteran Interview Guide:

Background Information

1. I wonder if you could start by telling me a little about yourself:

- Where were you born and raised?
- Age
- Branch of the military and years served

1. What has been your experience with the criminal justice system?

- Length of last incarceration
- Overview of prior incarcerations, including facilities and length of stay

Reentry and Linkage to Care Questions (constructs from CFIR and Behavioral Model in **bold**)

*I’d like to now ask a few questions about your most recent experience with incarceration, focusing primarily on the programs and services you received while in prison/jail and upon release to support your transition back to the community.*

1. Prior to being released this last time, what was done to help prepare you for transitioning back into the community?

- People you met with to discuss resources and needs
- Transition planning (housing, health care, mental health care, substance abuse treatment, etc)
  - Was he given his medical record? Was info shared with VA providers pre-release?
- Other supportive services?

1. What were some of the most important resources you thought you would need upon release?
2. What people or services did you find most useful when you were planning for your release? Why?
   1. Were there people or services that were not very useful? Why not?
3. What else do you think would be important to do prior to being released to help a Veteran transition back into his/her community?
4. Tell me about your experience transitioning back home to your community. How has it been?
5. How long have you been out?
6. What are you most proud of?
7. What has been the most challenging thing for you?
8. You mentioned earlier that some of the most important resources you thought you would need were [x,y,z]. What has been your experience trying to secure or obtain these resources?
9. Are there other services or resources that you have found you need now that you are out?
   - Health care
   - Mental health care
   - Substance abuse treatment or recovery support
   - Housing
   - Social support
   - Employment
10. To what extent have you been able to access these services or resources?
    1. What has been helpful for you?

*Probes:* relationship to probation officer; pre-release planning; linkage to care; relationship with community treatment provider; social situation

- 1. What has been most challenging?

*Probes*: relationship to probation officer; pre-release planning; linkage to care; relationship with community treatment provider; social situation; juggling multiple issues/concerns

- 1. Can you imagine a resource or service that would help you with some of these challenges?

1. (For sex offenders) How do you think your sex offender status has impacted your re-entry?

Networking Questions

1. What kind of social support do you currently have in your life?

*Probe:* Family, friends, sponsors, peers, others

1. What additional social support would be helpful for you?
2. Tell me about your experience interacting with people from VA. What are those interactions like?

Probes: easy/hard to contact; type of communication (e.g., phone, email, other); helpful; frustrating; attitudes towards you?

1. What programs (VA or community) or people (professionals, family, friends, peers) did you go to for help when you left prison? What were those experiences like?

Probes: easy/hard to get through; type of communication (e.g., phone, email, other); helpful; frustrating; follow-up; attitudes towards JIV?

1. How helpful are other people who had incarceration experience in giving you advice and tips about life after release from prison/jail? What kinds of advice did you receive from such people? (e.g. for help with housing, jobs, food, etc.) Did you get this advice while you were still incarcerated, after release, or both?
2. What other connections or resources would you like to be connected to? Why would they be beneficial to you?

Recommendations for peer support programs

1. We are developing a peer support service to help Veterans make the transition from prison to community settings. The peer would be a Veteran with incarceration experience. To what extent would it be helpful to you have peer support right now?
2. What do you imagine a peer could do that would be helpful for you and other Veterans with similar experiences?

*Probe:* Activities like going to appointments, helping establish connections within a community, providing information about programs/services, etc.

1. How often do you think it would be useful to meet with a peer?

Concluding Questions

1. What else do you think would be important for us to know about being a Veteran who is trying to get re-established within his/her community after incarceration?
2. Can I interview you again in [ __ weeks / ___ months] to see how things are going at that point?

[*Other Questions to Consider* – if not covered by the discussion so far -- added by KM 12/8/16]

- 1. What, specifically did you reentry plan look like?
     1. And what did it cover? (was it a list of medical/behavioral appointments? Was it address and contact person at housing (e.g. at GPD or at DOM, etc.).
     2. Was it a single piece of paper, or were there other resource materials attached?
     3. What was the process like to create the plan? (e.g. back and for the discussion about your needs and preferences, or more of just being told “This is your plan” without much opportunity for input)
     4. Who created it – a prison/jail employee or the person from the VA (e.g. Tom Palladino,
  2. What health appointments did you have set up when you came out? (which one(s) did you attend?)
  3. What behavioral health appointment did you have set up when you came out? (which one(s) did you attend?)

Stakeholder Interview Guide

Introduction: We are trying to understand the landscape of re-entry services in MA. Our ultimate goal is to improve re-entry services for Veterans. While VA has a national re-entry program, there has been less focus on follow-up services for Veterans in the few months after they return to their community. The VA has funded our group to design a program to better link Veterans with health care services after they are released. As part of this process we would like to better understand the context in which MA re-entry services currently operate and how Veterans fit into this structure. Do you have any questions before we get started?

1. Can you tell me a little bit about yourself and your role in this organization?
2. Can you tell me a little bit about your organization and the re-entry services that it provides?
   1. Tell me a little bit about the population that your organization works with
3. Can you walk me through the process/steps of what happens when you start working with a new client who is being released?
   1. How do they come to be involved with your organization?
   2. Do you work with clients before they are released?
   3. Do clients work with other organizations before, during, or after working with your organization?
4. Tell me about your collaborations with other organizations that serve re-entry clients?
   1. Are there some organizations you work more closely with than others? Which ones? How so?
5. Taking a step back, can you tell me a little bit about how re-entry services are structured in Massachusetts?
   1. Who are the players?
   2. Where does your organization fit in this picture?
6. Tell me about any gaps that you see in re-entry services in the state? What about gaps in the re-entry process for individual clients?
7. As you know, this project will result in an intervention targeting Veterans who are being released. Can you tell me how Veterans fit into the re-entry landscape?
   1. Services specific to Veterans? Organizations specific to Veterans?
   2. Any specific gaps in the system that Veterans might experience over other clients who are being released?
8. Can you describe the main challenges or barriers that your organization faces when assisting Veterans with re-entry issues. *(probe for health care-related issues)*
9. Tell me about your organization’s experience with the VA. Is there anything we should understand about these experiences or prior interactions?
10. As we begin to design an intervention, where do you think the greatest need is for Veterans as they re-enter their communities?
    1. How do we best engage with Veterans?
    2. How do you think a peer support intervention might work in this situation?
11. Is there anything else we should understand about re-entry services in the state or for Veterans in particular? What about for the Veteran population being released?
12. As part of this study, we would like to talk with additional stakeholders.
    1. Is there anyone else in your organization who would be critical for us to talk with?
    2. In the re-entry field in Massachusetts?
    3. Any of your program clients/graduates?
    4. Would your organization be interested in collaborating with us in the future as we design and implement this intervention?
